# Use of Bioelectrical Impedance Analysis to Explore the Effectiveness of Stellate Ganglion Block in Patients with Post-Stroke Complex Regional Pain Syndrome: A Retrospective Pilot Study

**DOI:** 10.3390/jpm14030258

**Published:** 2024-02-28

**Authors:** Jin-Whan Ryu, In-Su Hwang, Seung-Kyu Lim

**Affiliations:** Department of Rehabilitation Medicine, Soonchunhyang University Cheonan Hospital, Soonchunhyang University College of Medicine, Cheonan 31151, Republic of Korea

**Keywords:** electric impedance, complex regional pain syndromes, rehabilitation, autonomic nerve block, stroke

## Abstract

Post-stroke complex regional pain syndrome (CRPS) poses challenges in pain assessment for survivors. Stellate ganglion block (SGB) is a treatment, but evaluating its effectiveness is difficult for patients with communication limitations. Edema, a prominent symptom, can serve as an evaluation marker. Bioelectrical impedance analysis (BIA), assessing body composition and fluid status, is used independently of patient cooperation. This retrospective, observational pilot study aims to explore BIA’s utility as an assessment tool post-SGB, revealing the effects and time courses of a single SGB on the bodily composition of post-stroke CRPS patients. Seven patients received ultrasound-guided SGB with a 5 mL solution containing 4 mL of 0.25% bupivacaine hydrochloride and 40 mg of triamcinolone into the prevertebral muscle space. BIA compared measures between affected and unaffected arms. The affected arm had higher segmental body water (SBW) and extracellular water ratios before SGB (*p* = 0.028 and *p* = 0.018, respectively). The SBW of the affected side, the SBW ratio, and the 1 and 5 kHz SFBIA ratios improved over time (*p* = 0.025, 0.008, 0.001, and 0.005, respectively). Rapid improvement occurred around 3 days post-injection, with maximum effects within approximately 1 week, persisting up to 3 weeks. SGB successfully reduced edema in post-stroke CRPS patients, with BIA serving as a useful tool for follow-up, facilitating the development of efficient treatment plans.

## 1. Introduction

Post-stroke pain is common; it may cause functional and cognitive decline, fatigue, decreased quality of life, depression, and even suicidality in stroke survivors [1]. The various types of post-stroke pain include central post-stroke pain, spasticity-related pain, hemiplegic shoulder pain, complex regional pain syndrome (CRPS), and headache [1,2]. CRPS, previously known as reflex sympathetic dystrophy and causalgia, is subclassified according to the absence (type I) or presence (type II) of peripheral nerve injury [3]. Post-stroke CRPS constitutes type I; its reported prevalence is 8.94–61% [4,5]. The condition is also known as shoulder–hand syndrome because it usually involves the shoulder and wrist but spares the elbow [1]. The features of shoulder–hand syndrome include pain, swelling, edema, allodynia, hyperalgesia, limited ranges of motion of the shoulder, wrist, and hand joints, and a sense of hotness and redness in the wrist and hand [1]. In acute rehabilitation units, post-stroke CRPS hinders functional recovery.

Sympathetic blockade (SB) is widely used to treat CRPS type I because sympathetic nervous system dysfunction has been implicated in the pathogenesis of this condition [6]. The stellate ganglion is a component of the cervical sympathetic chain; it forms via the fusion of the inferior cervical ganglion to the first thoracic sympathetic ganglion at the level of C7 [7]. The ganglion delivers sympathetic inputs to the ipsilateral upper extremities; thus, stellate ganglion block (SGB) has been used to treat post-stroke CRPS in the upper limbs, leading to improvements in pain, swelling, range of motion, and functional activity [8,9,10].

No standard SGB protocol is currently available. Published protocols differ in terms of the drugs used, the amounts injected, the number of repeat injections, and the times between treatments. When evaluating effects, pain is the principal indicator. However, this is subjective; pain may not be accurately reported by stroke patients with poor verbal skills or cognitive decline or by patients who exhibit poor cooperation with medical personnel [11]. In contrast, swelling is both objective and grossly evident. Bioelectrical impedance analysis (BIA) is commonly used to estimate bodily composition and fluid compartment status. It provides highly sensitive quantification of edema regardless of patient cooperation [12]. BIA can also be used to measure volumes in post-stroke CRPS patients with edema or swelling of the upper extremities. If the extent of edema before and after SGB can be identified via BIA, treatment planning can be expedited, improving the number of injections and intervals between those injections. Therefore, the primary objective of this study was to explore the effectiveness of BIA as an assessment tool after SGB by observing changes in edema. Furthermore, our goal was to ascertain whether BIA could detect the effects and time courses of a single SGB on the body composition of patients with post-stroke CRPS.

## 2. Materials and Methods

### 2.1. Study Design and Participants

This retrospective, observational pilot study obtained approval for exemption from review and consent from the Institutional Review Board of Gyeongsang National University Changwon Hospital. Medical records were reviewed for seven patients with post-stroke CRPS who had been transferred to the rehabilitation unit from the stroke unit after acute stroke management from July 2020 to December 2021. Confirmation of CRPS in stroke patients is difficult using only clinical signs and symptoms; confounders include cognitive decline assessing through the Mini-Mental State Examination (MMSE) test [13], limited verbal expression, neglect, and motor weakness. The degree of disability or dependence in the daily activities of stroke patients was assessed using the modified Rankin Scale (mRS) [14]. A three-phase bone scan has been used to support a diagnosis of post-stroke CRPS in a previous study [15]. Asymmetrically increased uptake in the delayed phase (phase 3) of the affected limb is consistent with CRPS [15]. Thus, post-stroke CRPS was defined by the presence of suspect signs and symptoms in the hemiplegic, unilateral upper extremity using the Budapest criteria recommended by the International Association for the Study of Pain [16], combined with three-phase bone scan data. Three-phase bone scan images were obtained after the injection of 20 mCi Tc99m-hydroxydiphosphonate; these images were analyzed by a nuclear radiologist. The exclusion criteria were a bilateral hemispheric lesion; any condition that could trigger limb edema (heart or renal failure; the use of steroids, antidiuretics, or hormonal agents); and a history or injury of, trauma to, or surgery on the upper limb of the paralyzed side.

### 2.2. Intervention

**Figure 1:**
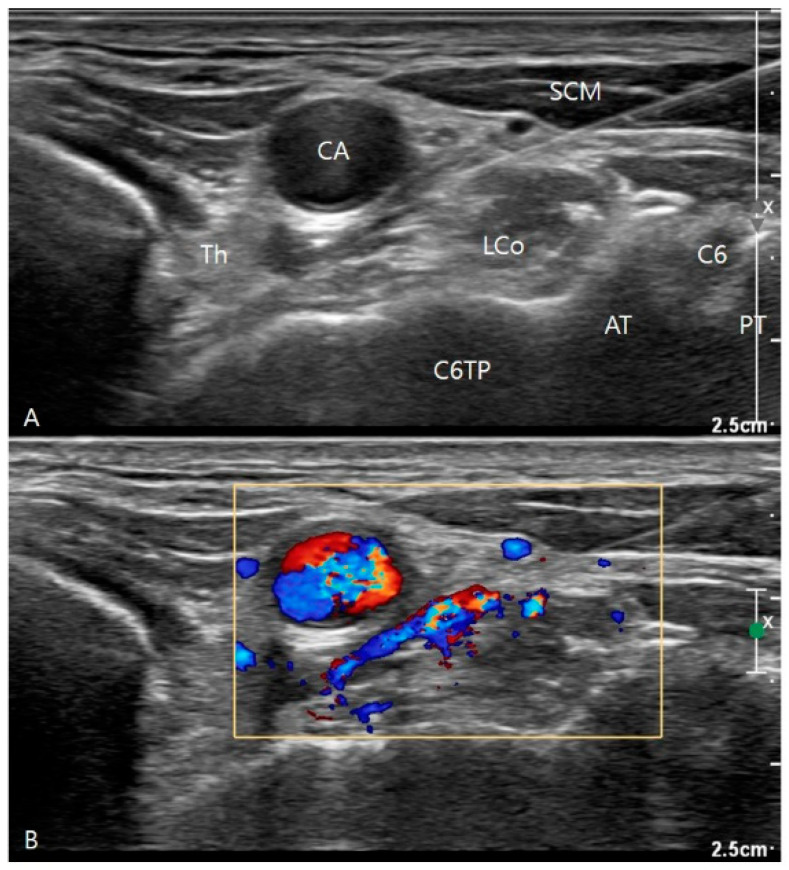
Transverse ultrasound image of the neck at the level of C6 for a left stellate ganglion block. (**A**) The needle tip was advanced to a position anterior to the longus colli muscle. (**B**) The power Doppler imaging showed the spread of the injectate over the prevertebral space of the muscle. CA, carotid artery; SCM, sternocleidomastoid muscle; Th, thyroid gland; LCo, longus coli muscle; C6TP, C6 transverse process; AT, anterior tubercle; PT, posterior tubercle.

The SGB protocol performed in post-stroke CRPS patients is as follows. After confirming post-stroke CRPS, a single SGB was conducted for each patient under ultrasound guidance (Affiniti 50, Phillips, Amsterdam, Netherlands). Each patient was placed in a supine position with the neck slightly extended and the head slightly rotated contralateral to the approached side. Due to the increased risk of side effects, such as vascular puncture, especially in stroke patients using antiplatelet agents, the C6 level was chosen as the block site to minimize potential complications in our rehabilitation unit [7]. The long axis of a linear probe (5–12 MHz) was placed horizontally to the plane at the level of C6 after identification of the C6 transverse process and its anterior and posterior tubercles; the probe was also located medial to the longus colli muscle and the prevertebral fascia. A 24-gauge 6 cm needle was inserted 1 cm from the probe using the in-plane technique. The tip of the needle was advanced to a position anterior to the longus colli muscle [17]. After aspiration had stopped at this position, 5 mL of liquid (including 4 mL of 0.25% (*w*/*v*) bupivacaine hydrochloride and 40 mg triamcinolone) were injected and spread over the prevertebral space of the muscle (Figure 1). After needle withdrawal, the patient remained supine for 5 min to assess injected-side Horner syndrome status. All adverse effects during or after blockade were recorded.

### 2.3. Outcome Measures

Bodily composition was measured via tetrapolar BIA (InBody S10; Biospace, Seoul, Korea) using a standardized procedure administered by a trained nurse. Patients removed all metallic objects in contact with the skin to avoid erroneous measurements; they were also instructed to avoid excessive fluid intake. During each procedure, a patient was placed supine with the arms and legs abducted from the body. BIA measures included segmental body water (SBW), phase angle (PhA), extracellular water (ECW) ratio, and single-frequency bioelectrical impedance analysis (SFBIA) of the upper extremities. The PhA indicates cellular health because it reflects changes in fluid balance, cellular membrane integrity, and cellular function [12]. Lower PhAs are associated with older age, malnutrition, volume overload, inflammation, infections, and systemic diseases [12]. The impedance (R) is inversely proportional to segmental volume [18]. Impedances were obtained at 1, 5, and 50 kHz because the PhA is maximal at 50 kHz and low-frequency (1 and 5 kHz) currents do not penetrate the cell membrane; instead, they pass through extracellular fluid and allow the measurement of extracellular volume (a lower score indicates a higher ECW ratio) [18]. The PhA was derived using a published predictive formula: PhA (°) = arctan (Xc/R) × (180/π) [19]. The difference between, and the ratio of, the values of both arms were used to compare the two sides. The ΔSBW, as well as the ratios of SBW, PhA, segmental ECW, and SFBIA, were calculated as follows:

ΔSBW = affected SBW − unaffected SBW;

SBW ratio = affected SBW/unaffected SBW;

PhA ratio = affected PhA/unaffected PhA;

ECW ratio affected = affected ECW/total body water;

ECW ratio (inter-limb, I) = affected ECW ratio/unaffected ECW ratio;

SFBIA ratio = unaffected SFBIA/affected SFBIA [20].

BIA assessments were conducted before SGB and immediately after the injection, at the midpoint of the first week, and at the end of the first week to observe acute changes. Subsequently, to assess sustained effects, evaluations were conducted at 2 and 3 weeks after the injection. Although pain intensity is an important outcome, it cannot be evaluated in patients with aphasia or cognitive decline.

### 2.4. Statistical Analysis

The baseline clinical and bodily composition data are presented as means with standard deviations for continuous variables and as proportions for categorical variables. The Wilcoxon signed-rank test was used to compare the baseline bodily compositions of affected and unaffected arms. The Friedman test was used to compare BIA-assessed variables at various time points. Post hoc analysis comparing pre-injection and all post-injection data was conducted using the Wilcoxon signed-rank test with Bonferroni correction. All statistical analyses were performed using IBM SPSS Statistics ver. 24.0 software (IBM Corp., Armonk, NY, USA). *p*-values < 0.05 were considered statistically significant.

## 3. Results

The mean patient age was 65.9 ± 7.2 years. The hemiplegic side was the right side in five (71.4%) patients and the left side in two (28.6%) patients; all patients were right-handed. Among the seven patients, five could not adequately describe pain intensity or characteristics because of aphasia, cognitive decline, or irritability. Four patients exhibited shoulder subluxation of the affected arm. Post-stroke CRPS was diagnosed via a three-phase bone scan at a mean of 39.3 ± 19.3 days after stroke; SGB was performed at a mean of 44.6 ± 16.9 days after stroke. The baseline characteristics of all patients are shown in Table 1.

The baseline bodily composition data were analyzed using BIA (Table 2). Comparative analysis of both arms revealed that the SBW and ECW ratios before SGB were significantly higher in the affected arm (*p* = 0.028 and *p* = 0.018, respectively). The PhA, as well as the 1 and 5 kHz SFBIAs before SGB, were significantly higher in the unaffected arm (all *p* = 0.018).

**Figure 2:**
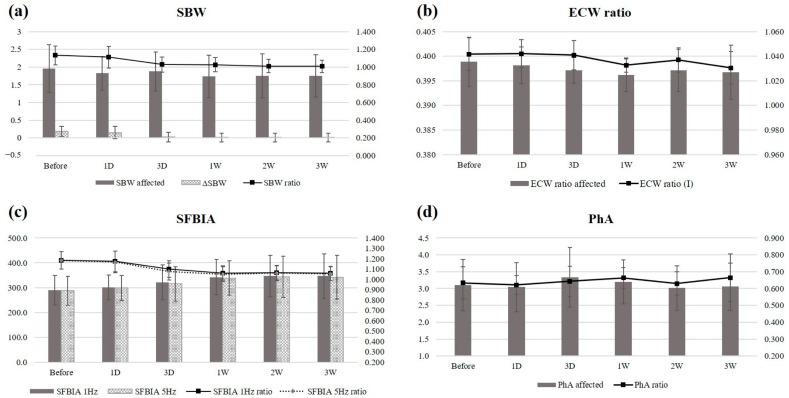
Time-dependent changes in (**a**) SBW, (**b**) ECW ratio, (**c**) SFBIA, and (**d**) PhA after a single SGB. SBW, segmental body water; SFBIA, single-frequency bioelectrical impedance analysis; ECW, extracellular water; PhA, phase angle; I, inter-limb.

The SBW of the affected side, the SBW ratio, and the 1 and 5 kHz SFBIA ratios improved over time (*p* = 0.025, 0.008, 0.001, and 0.005, respectively) (Figure 2). Post hoc analysis revealed significant differences between pre-injection and post-injection values, and the parameters rapidly improved approximately 3 days after each injection. The maximum effects were achieved within approximately 1 week and persisted thereafter.

Neither the ECW ratios nor PhA values exhibited consistent patterns during follow-up. In one patient, the effect of SGB was maximal between days 3 and 7, then decreased. Although the time courses of improvements in the ECW ratio and PhA were different from other parameters, all BIA parameters improved from baseline by 3 weeks after injection. No other adverse event occurred in patients at risk. Pain was not consistently measured accurately in all patients, but improvements in pain after the injection were sometimes noticeable when observing nonverbal responses or expressions.

## 4. Discussion

To our knowledge, this study is the first to use BIA to investigate the effectiveness of an SGB and the time course of its effects on the bodily compositions of patients with post-stroke CRPS. BIA revealed improvements over 3 weeks after a single SGB of the SBW of the affected side. The SBW, 1 kHz SFBIA, and 5 kHz SFBIA ratios revealed the duration of SGB persistence.

Post-stroke CRPS is a serious disabling complication in stroke survivors. Although the pathogenesis remains unclear, the proposed mechanisms include impaired biomechanics of the glenohumeral joint (i.e., subluxation, motor weakness, and immobility of the upper extremity), nociceptive sensitization, immune system changes, and autonomic dysregulation [2]. The prevalence of post-stroke CRPS varies according to the population studied and the diagnostic criteria, but it affects 30% to 65% of stroke patients and usually manifests within 3 months after stroke [4,5]. Although the post-CRPS incidence was not identifiable in this study, post-stroke CRPS developed in the subacute post-stroke period (39.3 ± 19.3 days after stroke). After appropriate acute stroke management, rehabilitation during this phase is important because 95% of stroke survivors achieve their best possible neurological levels within 11 weeks of onset; however, individuals who have experienced severe strokes require a mean interval of 15 weeks to achieve that level [21]. Motor recovery and learning after stroke reflect re-organization of the cerebral cortex; they are attributable to intensive task-specific practice [22]. Post-stroke CRPS itself adversely affects patients; it also promotes inactivity and hinders manual rehabilitation. Therefore, if patients with post-stroke CRPS do not receive appropriate rehabilitation because the CRPS is not correctly managed, functional recovery may be compromised, impairment may develop, and quality of life may be reduced. It is important to treat post-stroke CRPS in acute-to-subacute rehabilitation settings.

Various treatments for CRPS have been proposed; there remains no definitive treatment for post-stroke CRPS. Management should focus on functional restoration [1]. An integrated approach involving pharmacological and non-pharmacological modalities to control pain and edema, mobilize and strengthen the affected limb, and achieve desensitization is required by patients with post-stroke CRPS [1]. SGB is commonly used to treat this condition. SGB interrupts the positive feedback circuit, decreases central hyperexcitability, and regulates nociceptive effects by blocking sympathetic input to the ipsilateral upper extremity, chest, face, and head [23]. Several reports have appeared regarding the efficacy of various SGB protocols in patients with CRPS type 1 [8,9,10]. Yucel et al. [9] compared the effects of SGB according to symptom duration (17.0 ± 6.3 weeks vs. 49.8 ± 17.6 weeks) in 42 patients with CRPS type 1. SGB with 15 mL of 0.5% (*w*/*v*) bupivacaine (5 mg/mL) and 1% (*w*/*v*) prilocaine hydrochloride (20 mg/mL) was performed weekly for 3 consecutive weeks in both groups. Patients with shorter disease durations exhibited significantly better outcomes in terms of pain relief and range of motion improvements at 2 weeks after injection. Yoo et al. [8] reported that, at 2 and 4 weeks after blockade, ultrasound-guided SGB was more effective than blind injections in terms of pain relief, hand volume reduction, and CRPS score in 42 patients with post-stroke CRPS. In that study, 5 mL of 0.5% (*w*/*v*) lidocaine was injected twice at an interval of 1 week. In the retrospective case series reported by Wei et al. [10], ultrasound-guided SGB was used to deliver 5 mL of bupivacaine 0.5% *w*/*v* per patient at intervals of 2 and 7 days, combined with occupational and pharmacological therapies. Thus, the efficacy of combined treatment was explored.

It is challenging to definitively evaluate SB effectiveness with respect to controlling pain severity and duration in CRPS patients because there have been few high-quality trials, and there is no standardized SB protocol. Local anesthetics (LAs), such as bupivacaine and lidocaine, are commonly used to induce SGB, with or without steroids. Price et al. [24] performed SGB and lumbar SB using 15 mL of 1% *w*/*v* lidocaine or 15 mL of normal saline, then evaluated the diagnostic and therapeutic utilities of SB with LAs in seven CRPS patients. The peak analgesic effects developed 30 min after both blockades; the mean analgesia durations were 12 h and nearly 6 days for the saline block and SB with LAs, respectively. Additionally, LAs such as lidocaine and bupivacaine extended the duration of pain relief by 3–5 days, compared to placebo. Carroll et al. reported an increased duration of relief using botulinum toxin A (75 units) for lumbar sympathetic ganglion blockade, compared to bupivacaine alone (10 mL of 0.5% *w*/*v* bupivacaine) (71 vs. <10 days) [25].

In a rat model of CRPS, noradrenaline released from sympathetic nerve terminals triggers the production of an inflammatory mediator (interleukin-6) by epidermal keratinocytes; noradrenaline binds to β_2_-adrenoreceptors [26]. α_1_-adrenoreceptors are upregulated in the skin of CRPS-affected limbs in humans, potentially increasing the effects of sympathetic activation [27]. Corticosteroids exert anti-inflammatory effects and block nociceptive inputs; they also act as weak LAs and membrane stabilizers [28]. Similar to the mechanism of LAs, corticosteroid activation of the sodium pump triggers membrane hyperpolarization, thus stabilizing the membrane and preventing the hyperexcitation of abnormally excitable sympathetic nerve terminals [29]. SB with LAs and steroids may reduce sympathetic outflow and increase circulating levels of noradrenaline and α_1_-adrenoreceptors via similar pharmacological actions [30]. In a study investigating the effects of SGB on breast cancer-related lymphedema and corticosteroid efficacy, the arm circumferences significantly decreased in groups that received higher doses of triamcinolone (40 mg vs. 20 mg and 0 mg, with 5 mL of 0.5% bupivacaine). It has been suggested that corticosteroids enhance SGB-mediated immune modulation [31]. In this study, a single ultrasound-guided SGB using a mixture of 4 mL 0.25% (*w*/*v*) bupivacaine hydrochloride and 40 mg triamcinolone was associated with improvement at approximately 3 days after injection, which became maximal at approximately 1 week and persisted for 3 weeks. Although our protocol and outcomes differ from the methods and findings in previous studies, a single SGB with low doses of LA and a steroid exhibited longer-lasting effects than other blockades in previous studies. It remains unclear why a mixture of an LA and a steroid provides long-lasting effects, but volume reduction lasted longer than the few hours of LA effects. Our results may be attributable to the combined use of a steroid and ultrasound guidance, along with the short duration of post-stroke CRPS. Although further research is needed, our SGB time course in patients with post-stroke CRPS may enable the establishment of more efficient and effective treatment plans.

In post-stroke CRPS patients, it is challenging to assess pain using conventional methods (e.g., the visual analog scale) because verbal expression is often limited in relation to language problems or cognitive impairment. Edema can then serve as the primary outcome after injection. SGB appears to reduce edema by improving local vasodilation and lightening the fluid load on lymphatic capillaries; the sympathetic nervous system is presumed to directly regulate lymphatic flow [32]. In a previous study, SGB has been used to treat breast cancer-related lymphedema, which exhibits peripheral edema similar to the symptoms of CRPS [33]. Additionally, SB or neurolysis reduces the edema caused by CRPS [34]. Edema can be quantitatively evaluated by measuring volumes and circumferences. However, these methods depend on the quality of the examiner and require patient cooperation. In contrast, BIA is a portable, simple, reproducible, non-invasive, and widely used method that estimates bodily composition and fluid compartment status; patient cooperation is not required [12]. BIA measures bodily resistances and reactances by determining the electrical impedances when various electrical currents pass through different tissues [12,18]. BIA yields raw impedance parameters, hydration status, and body cell masses and cell integrities [12,18]. BIA can be repeatedly performed before and after treatments or interventions. BIA measures both segmental and whole-body compositions; thus, it enables comparisons of affected and unaffected limbs.

The SBW, ECW ratio, and SFBIA parameters reflect hydration status. The SBW is the sum of the segmental intracellular water and ECW values in all limbs. The ECW ratio is the ratio of the total body water to the ECW. Impedance refers to opposition to the flow of a current passing through the body and is inversely proportional to the amount of water present [18]. The SFBIA values at low frequencies (1 and 5 kHz) have been used to evaluate the ECW because such currents do not pass through cell membranes [18,35]. These parameters reflect ECW accumulation. The PhA is the phase shift between current and voltage triggered by an electrochemically charged membrane [12]. This parameter reflects changes in fluid balance, cellular membrane integrity, and cellular function; it also indicates cellular health, as well as nutritional and functional statuses [12]. PhA is affected by age, sex, and body mass index; it is frequently lower than normal in patients who exhibit several different diseases associated with infection, inflammation, or disease-specific perturbations [12]. In this study, the SBW, as well as the SBW, 1 kHz SFBIA, and 5 kHz SFBIA ratios, of the affected arm improved over time; neither the ECW ratio nor the PhA consistently improved during follow-up. One patient exhibited a unique time course of rapid improvement at approximately 3 days after injection, which diminished at approximately 1 week. We cannot explain why this patient differed from others. The data of this patient increased the standard deviations and thus considerably influenced the results. Notably, the PhA may not adequately assess edema because the PhA is affected by many conditions associated with neurological changes in the hemiplegic limbs of stroke patients. Further studies with additional patients are required to explore the effect of SGB on bodily composition and the clinical significance of the BIA parameters. However, BIA parameters can be used to evaluate the effect of SGB by measuring the extent of edema.

Our study had some limitations. First, it is difficult to form definite conclusions considering the retrospective design, the small number of patients, and the short follow-up period. There was no evidence supporting the standard use of BIA for SGB, and proper evaluation was challenging due to issues such as holidays, equipment availability, and hospitalization periods. Consequently, the number of patients who were regularly and consistently assessed was very limited. Second, the prevalence of aphasia or cognitive decline in the majority of patients limits the assessment of pain intensity, hindering the confirmation of SGB effectiveness. Additionally, we could not confirm the duration and extent of symptom relief provided by an SGB because we lacked control groups treated with a placebo or LA alone and did not inject repeatedly. Future studies should include larger sample sizes, longer-term follow-ups, and comparisons with various control groups. These aspects would enhance the evidence regarding SGB’s effectiveness.

## 5. Conclusions

The findings of this pilot study support the hypothesis that SGB effectively reduces edema in patients with post-stroke CRPS. Despite several limitations in the study design and the number of patients, BIA can serve as a useful assessment tool during follow-up after injection by observing changes in edema, potentially facilitating the development of efficient treatment plans.

## Figures and Tables

**Table 1:** Patient characteristics.

Variables	Patients (*n* = 7)
Age (years)	66 [63–66.5]
Sex (male:female)	4 (57.1):3 (42.9)
Type of stroke, infarction:hemorrhage	4 (57.1):3 (42.9)
Hemiparetic side, right:left	5 (71.4):2 (28.6)
Dominant side, right:left	7 (100):0 (0)
Modified Rankin Scale	
0–2	0 (0)
3	1 (14.3)
4	6 (85.7)
MMSE	11.5 ± 9.0
Aphasia	1 (14.3)
Neglect	1 (14.3)
Shoulder subluxation	4 (57.1)
Time to diagnose CRPS, day	32 [26–54.5]
Time to perform SGB, day	36 [33–55.5]

Values are expressed as median [interquartile range] or number (proportion). MMSE, Mini-Mental State Examination; CRPS, complex regional pain syndrome; SGB, stellate ganglion block.

**Table 2:** Baseline bioelectrical impedance analysis.

Variables		Values	
Height (cm)		163 [161.5–171.5]	
Body weight (kg)		65.5 [58.05–74.55]	
BMI (kg/m^2^)		24 [22.5–24.5]	
SMM (kg)		24.1 [20.05–29.9]	
PBF (%)		24.8 [22.35–30.8]	
FFM (kg)		45.1 [38.55–54.95]	
TBW (L)		33.5 [28.55–40.85]	
ICW (L)		20 [16.9–24.5]	
ECW (L)		13.5 [11.65–16.4]	
SBW (L)	Affected arm	1.66 [1.46–2.35]	*p* = 0.028
	Unaffected arm	1.59 [1.17–2.34]	
	Difference	0.22 [0.05–0.3]	
	Ratio	1.14 [1.03–1.23]	
ECW ratio	Affected arm	0.399 [0.395–0.405]	*p* = 0.018
	Unaffected arm	0.384 [0.373–0.389]	
	Inter-limb ratio	1.041 [1.029–1.057]	
Phase angle	Affected arm	3.2 [2.55–3.5]	*p* = 0.018
	Unaffected arm	4.9 [4.2–5.3]	
	Difference	1.9 [1.45–2.15]	
	Inter-limb ratio	0.57 [0.55–0.72]	
SFBIA 1 kHz	Affected arm	273.4 [271.6–312.5]	*p* = 0.018
	Unaffected arm	344.1 [293.55–378.1]	
	Ratio	1.192 [1.086–1.264]	
SFBIA 5 kHz	Affected arm	274.7 [268.8–310.05]	*p* = 0.018
	Unaffected arm	336.2 [292.2–375.9]	
	Inter-limb ratio	1.205 [1.084–1.258]	

Values are expressed as median [interquartile range] or number (proportion). BMI, body mass index; SMM, skeletal muscle mass; PBF, percentage of body fat; FFM, fat-free mass; TBW, total body water; ICW, intracellular water; ECW, extracellular water; SBW, segmental body water; SFBIA, single-frequency bioelectrical impedance analysis.

## Data Availability

The data presented in this study are available on request from the corresponding author.
